# The effectiveness of treatment for Severe Acute Malnutrition (SAM) delivered by community health workers compared to a traditional facility based model

**DOI:** 10.1186/s12913-018-2987-z

**Published:** 2018-03-27

**Authors:** J. L. Alvarez Morán, G. B. Franck Alé, P. Charle, N. Sessions, S. Doumbia, S. Guerrero

**Affiliations:** 1 Action Against Hunger UK, First Floor, Rear Premises, 161-163 Greenwich High Road, London, SE10 8JA UK; 2 Area of Preventive Medicine and Public Health, Rey Juan Carlos University, Avda. Atenas s/n, 28922 Alcorcón, Madrid Spain; 3 Fundación Acción Contra el Hambre | ACF-Spain, C/ del Duque de Sevilla 3, 28002 Madrid, Spain; 4 Action Against Hunger UK, First Floor, Rear Premises, 161-163 Greenwich High Road, London, SE10 8JA UK; 5 Faculty of Medicine and Odontostomatology, University of Sciences, Techniques and Technology of Bamako, BP 1805 Bamako, Mali; 6 Action Against Hunger USA, One Whitehall St, New York, NY 10004 USA

**Keywords:** Community-based Management of Acute Malnutrition (CMAM), Severe acute malnutrition (SAM), Coverage, Community health workers, Child nutrition

## Abstract

**Background:**

In most health systems, Community Health Workers (CHWs) identify and screen for severe acute malnutrition (SAM) in the community. This study aimed to investigate the potential of integrating SAM identification and treatment delivered by CHWs, in order to improve the coverage of SAM treatment services.

**Methods:**

This multicentre, randomised intervention study was conducted in Kita, Southwest Mali between February 2015 and February 2016. Treatment for uncomplicated SAM was provided in health facilities in the control area, and by Community Health Workers and health facilities in the intervention area. Clinical outcomes (cure, death and defaulter ratios), treatment coverage and quality of care were examined in both the control and intervention group.

**Results:**

Six hundred ninety nine children were admitted to the intervention group and 235 children to the control group. The intervention group reported cure ratios of 94.2% compared to 88.6% in the control group (risk ratio 1.07 [95% CI 1.01; 1.13]). Defaulter ratios were twice as high in the control group compared to the intervention group (10.8% vs 4.5%; RR 0.42 [95% CI 0.25; 0.71]). Differences in mortality ratios were not statistically significant (0.9% in the intervention group compared to 0.8% in the control group). Coverage rates in December 2015 were 86.7% in intervention group compared to 41.6% in the control (*p* < 0.0001).

**Conclusions:**

With minimal training, CHWs are able to appropriately treat SAM in the community. Allowing CHWs to treat SAM reduces defaulter ratios without compromising treatment outcomes and can lead to improved access to treatment.

**Trial registration:**

Retrospectively registered in ISRCTN Register with ISRCTN33578874 on March 7th 2018.

## Background

Severe Acute Malnutrition (SAM) is a global public health issue that affects an estimated 16 million children under the age of five worldwide [1] and is associated with an estimated 1–2 million deaths a year, though recent evidence suggests that this number might be significantly lower [2–4]. Previously, SAM was managed on an inpatient basis in hospital settings resulting in good clinical outcomes. However, the centralised nature of hospitals, resulting high opportunity costs for caregivers as well as far distances to access care, and the risk of cross infection led to treatment coverage rates as low as 10% being reported [5–7]. Given these factors, the Community-based Management of Acute Malnutrition (CMAM) model, previously known as Community based Therapeutic Care (CTC) approach, was introduced in 2000 enabling children with SAM to be treated closer to their homes [8]. This shift in paradigm has led to a rapid increase in the number of children receiving treatment, yet up to 80% of all children suffering from SAM are still unable to access treatment [5].

The capacity to meet global SAM needs is dependent on treatment coverage being significantly improved [9] by addressing barriers to access. Common barriers include lack of awareness of malnutrition services at a community level, high opportunity costs, and distance to treatment services [10]. As a means of improving coverage of other health interventions, task shifting of services to community health workers has been explored. For example, integrated Community Case Management (iCCM), a strategy to extend case management of childhood illness beyond health facilities so that more children have access to lifesaving treatments, has shown high treatment coverage and high quality care rates for sick children under five [11, 12]. The iCCM package differs across different contexts, but most commonly include diarrhoea, pneumonia and malaria interventions. The iCCM package also included the identification and referral of children with SAM by CHWs but does not currently include treatment of SAM at a community level [13].

In Mali, outpatient treatment of SAM has been delivered by trained Health Workers from Primary Health Facilities throughout the country but the high malnutrition rates and difficult geographical access hinders the impact of treatment. The latest prevalence data show a Global Acute Malnutrition (GAM) rate in Kayes region of 13.3% (95% CI: 10.9% - 16.1%) and Severe Acute Malnutrition (SAM) rate of 2.4% (95% CI: 1.6% -3.6%). Coverage assessments carried out in March 2013 found treatment services admitting an estimated 24.9% (95% CI: 14.5% - 39.2%) of all children suffering from SAM [14].

Kita has two layers of community health staff- a cadre of unpaid community volunteers (Relais Communautaire, in French) and a salaried network of Community Health Workers (Agent de Santé Communautaire, in French). The CHWs provide integrated management of malaria, diarrhoea and pneumonia as well as screening, referral and follow-up of children with malnutrition. Community Health Workers (CHWs) are based at community level and managed through a decentralized apolitical and social model (ASACO, Association de Santé Communautaire) integrated into the health system. CHWs offer iCCM which is known as SEC services in Mali (Soins Essentielle dans la communauté) for malaria, pneumonia, diarrhoea and nutrition activities, such as screening and counselling. These integrated community services have been implemented in Mali since 2012 [15]. CHWs in Mali are therefore well positioned, as is the case in other countries, to potentially deliver acute malnutrition treatment services.

A few studies have investigated the possibility of treating SAM at a community level and assessed such potential within research settings [16, 17]. However, to our knowledge, this is the first time that a pilot has been conducted of such an approach, in real operational field conditions.

The purpose of this study was to explore the potential of integrating SAM treatment as part of iCCM services delivered by CHWs. It hypothesised that the integration of SAM treatment as part of the iCCM package, delivered by CHWS, would provide earlier identification of SAM cases, better access to treatment and improved clinical outcomes (including cure, death and defaulter ratios). The study was conducted between February 2015 and February 2016 in the communes of Tambaga, Bougarabaya and Kobiri in the area of Kita, in the Kayes region of Mali.

## Methods

This multicentre, randomised intervention study compared two groups of children with SAM in neighbouring sectors of Kita district, and followed outcomes for a period of 12 months. The control group received outpatient treatment for uncomplicated SAM from health centres (4), whilst the intervention group received outpatient treatment for uncomplicated SAM from health centres (3) or Community Health Workers. The allocation of treatment between the two groups was randomized and rationalized. Comparability of both groups was ensured by identifying key indicators in a transversal sociodemographic baseline survey implemented prior to the intervention. The survey examined population under 5 years old, economic characteristics, availability and quality of health services, costs and utilisation of treatment services, population type (nomadic or sedentary), sanitation and social characteristics of both groups. The survey was a cluster-randomised sample with two stages: first clusters/villages were selected proportional to their population size. In each cluster, the modified EPI method was then used to select the households. During this survey, prevalence of SAM was also measured (see Table 1).

**Table 1:** Baseline study of sociodemographic characteristics

Class	Indicator	Control		Intervention		*P* value
		n (mean)	% (SD)	n (mean)	% (SD)	
Demo Graphics	Total population	1093		1311		NA
	Sex (male)	552	50.5%	671	51.2	NA
	Population < 5 years	230	21.0%	302	23.0%	NA
	GAM by MUAC	11	5.0%	10	4.3%	0.717
	Households surveyed (*n*)	120		119		
Living conditions	Improved sanitation	43	35.8	45	38.1	0.713
	Concrete flooring	25	20.8	18	15.2	0.263
	Congregated iron roofing	99	82.5	40	33.9	< 0.001
	Access to clean water	98	81.7	62	52.5	< 0.001
	House ownership	114	95.0	110	93.1	0.560
Socio economic status	Low	48	40.0	48	40.7	0.915
	Medium	24	20.0	23	19.5	0.922
	High	48	40.0	47	39.8	0.979
Health care access for child under 5	Caregiver responded to sick child	112	96.5	113	98.3	0.683
	Used Health Centre	106	94.6	99	90 .8	0.274
	Used Traditional medicine	41	36.6	47	43.1	0.339
	Used Self-medication over the counter	7	6.2	11	10.1	0.297
	Used self-medication with pharmacies visit	8	7.1	3	2.7	0.133
	Cost of consultation^a^	(489)	(342)	(358)	(420)	0.060
	Cost of medicines^a^	(5678)	(3429)	(6267)	(3088)	0.310

^a^Out of pocket expenditure for children under five years old in the last 6 months in francs CFA. Mean and Standard Deviation

Selection criteria for both groups included: child meeting the definition of having SAM according to Mali’s national protocol (i.e. aged between 6 and 59 months; Middle Upper Arm Circumference (MUAC) < 115 mm; Bilateral oedema or Weight for Height (WHZ) < − 3 Z-score), and parental consent given to take part in the study. In both locations, the study evaluated four key sets of indicators: clinical outcomes (cure, death and defaulter ratios) of children enrolled in the programme, cost effectiveness, treatment coverage and quality of care. Two further indicators were considered relevant and collected: MUAC at admission and cases referred to hospitalization on the first day of treatment.

Prior to the initiation of the study, baseline data on all four indicators was collected. National and district level meetings were conducted with key stakeholders in order to outline the objectives of the study and to promote greater engagement of the health authorities in both the implementation of activities and utilisation of results.

CHWs within both groups were initially trained for 2 weeks on iCCM and CMAM and received refresher training 6 months into the study. They were required to comply with Malian protocols for iCCM and CMAM using MUAC, WHZ and oedema for admission into nutrition treatment and checking for Vitamin A and deworming medication needs. They were all equipped with MUAC tapes, height-boards and weighing scales. As per national guidelines, CHWs were also able to prescribe and distribute antibiotics. Hygiene kits containing capsules for the treatment of water to each home admitted to the program were distributed. CHWs, with the support of community volunteers, carried out active community screening every 3 months and passive screening through the study period. CHWs referred all children with complicated SAM (i.e. presence of danger signs and failed appetite test) to a nearby Stabilisation Centre for inpatient care. Joint supervisory visits by Action Against Hunger and the National Institute for Research in Public Health (INRSP) were carried out in a standardised manner in both groups. The sociodemographic characteristics of CHWs are outlined in Table 2 as per data collected during a survey in the intervention area.

**Table 2:** Sociodemographic profile of CHWs (*n* = 17)

	Median (Min-Max)	Number	Percent
Age (years)	25(19–61)	–	–
Sex			
Male		4	23.5
Female		13	76.5
Marital Status			
Single		6	35.3
Married		10	58.8
Divorced		1	5.9
Education level			
Primary		3	17.6
Secondary		13	76.5
High		1	5.9
Education degree			
Midwife		13	76.5
Health Aide^a^		3	17.6
Other		1	5.9
Number of months of training received	6(3–12)	–	–
Number of years worked in the health sector	3(1–9)	–	–
Number of years worked as a CHW	3(1–5)	–	–
Total number of CHWs assessed during the study		17	100

^a^ Health staff who has received at least 6 months training in a health school and having passed an internship of 3 months in a health centre

Data was collected electronically in real time via smart phones and Open Data Kit (ODK) designed software [18]. A dedicated data manager performed data quality checks. Throughout the study, CHWs and clinic workers received supervision twice a month by the ACF staff and once every 3 months by the staff of National Institute for Research in Public Health (INRSP).

A critical indicator to evaluate the performance of nutritional programs is the proportion of children discharged as cured (cure ratio) since this is influenced by the quality of the care provided. This is defined as follows:

$$ Cure\ Rate=\frac{Cured\ Cases}{Cured\ Cases+ Deaths+ Defaulters+ Non\ Respondents}X100 $$

A cured case, according to national CMAM protocol, is a child with a Weight for Height Z score ≥ − 1.5 and absence of nutritional oedema during 14 days or a MUAC > 125 mm and absence of nutritional oedema during 14 days. Defaulter cases are defined as those who missed two consecutive appointments (14 days). Defaulter ratios and mortality ratios were similarly calculated as above. MUAC at admission was also collected in order to assess whether children accessed treatment “early” (114 mm–110 mm) or at “advanced” stages (< 110 mm) of the SAM episode.

Usual descriptive techniques were used; for quantitative variables, the mean and standard deviations were calculated. The economic well-being index of the household was constructed using Principal Component Analysis (PCA) based on asset data owned by households (information on household assets obtained by collecting information on households of certain consumer goods such as television, radio or car), scores were then divided by quintiles in the population and presented as 3 categories (low, medium and high). For comparison between zones, T student test was used within the baseline study. Aside from the descriptive techniques, analyses of Relative Risks adjusted for age, sex, edema and MUAC at admission and 95% Confidence Intervals were implemented and association between both strategies was assessed using χ^2^ test (with Yates correction when necessary). Null hypothesis of no effect were rejected at *P* ≤ 0.05. Analyses were implemented using R software.

The Semi-Quantitative Evaluation of Access and Coverage (SQUEAC) methodology was used to assess treatment coverage across both the control and intervention group. SQUEAC offers a reliable direct method of assessing coverage of CMAM programs, adapted to small areas, and provide a detailed analysis of programme barriers [19]. Coverage assessments were implemented in Kita in December 2014, before the intervention started, in June 2015 and in December 2015 (10 months after the start of the study). A single coverage indicator was used to estimate the coverage of treatment services, or the proportion of eligible cases who have received treatment [20]. A single coverage indicator takes into account recovering cases both within and outside of the programme using the following formula:

$$ Single\ Coverage=\frac{\mathrm{Cin}+\mathrm{Rin}}{\mathrm{Cin}+\mathrm{Rin}+\mathrm{Cout}+\mathrm{Rout}} $$

- Cin = Current SAM cases in the programme
- Cout = Current SAM cases not in the programme
- Rin = Recovering SAM cases in the programme
- Rout = Recovering SAM cases not in the programme

## Results

In total, 699 children were admitted to the intervention group and 235 children were admitted to the control group. ‘Matched pair’ analyses was used to mitigate the lower sample size in the control group. Almost 50% of cases were admitted based on MUAC and more children in intervention group reported MUAC measurements between 150 mm -115 mm at admission compared to control group (52.9% vs. 46.8%). There were significantly more children with oedema at admission in the control group compared to the intervention group (4.7% vs. 0.7%). More children in the intervention group tested positive for malaria (35.9% vs. 19.7%). 10% of cases were not tested for malaria in both the control and intervention group.

**Fig. 1:**
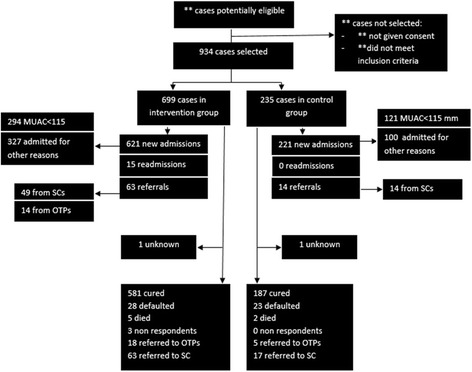
Flow diagram of cases

Figure 1 outlines the flow of admissions to treatment outcomes within both the intervention and control groups. Out of the 699 cases in the intervention group, 621 were new admissions in which the majority (52.7%) were admitted on criteria other than MUAC. Within the control group, 221 cases, out of 235, were new admissions in which the majority (54.8%) were admitted based on MUAC< 115 mm. Non-respondent figures were not calculated in the control group since national protocols at health centre level do not collect this indicator, thus they were recorded as zero. Hence, non-respondent ratios between the two groups cannot be compared. Furthermore, relapse ratios could only be measured in the intervention group as the Malian definition of relapse is imprecise and data routinely collected in the different health centres were not comparable. The analysis provides no indication on whether relapse ratios were affected by the provision of treatment by CHWs.

Table 3 outlines the characteristics of children at admission in both the intervention and control group. Groups were similar in terms of age breakdown and gender, with children aged between 6 and 24 months forming the majority of admitted cases. There was marginally more males admitted in the control group (46.8% compared to 41.1% in the intervention group) but this did not represent a significant difference.

**Table 3:** Characteristics of intervention (*n* = 699) and control group (*n* = 235) at admission

		Intervention Group		Control Group	
		*n*	%	*n*	%
Age (in months)	6–12	393	56.2	142	60.4
	12–24	235	33.6	77	32.8
	24+	71	10.2	16	6.8
Sex	Male	287	41.1	110	46.8
	Female	412	58.9	125	53.2
MUAC at admission	150- 115 mm	370	52.9	110	46.8
	114-110 mm	248	35.5	97	41.3
	109-106 mm	23	3.3	7	3.0
	< 105 mm	58	8.3	21	8.9
Presence of oedema	Yes	5	0.7	11	4.7
	No	694	99.3	224	95.3
Malaria test	Negative	409	58.5	157	66.8
	Positive	229	32.8	41	17.4
	Not tested	61	8.7	37	15.7
Type of admission	New	621	88.8	221	94.0
	Readmission	15	2.1	0	0.0
	Referral from SC^a^	49	7.0	14	6.0
	Referral from OTP	14	2.0	0	0.0
Place of admission	Health facility	147	21.0	235	100.0
	CHW	552	79.0	0	0.0
Referred by	CHW	260	37.2	21	8.9
	Volunteer CHW	138	19.7	141	60.0
	Caregiver (Mother)	258	36.9	58	24.7
	Other	43	6.2	15	6.4

^a^SC: Stabilization Centres are used for inpatient treatment of cases with complications. The majority of cases, once stabilized, are sent back to the community to continue the treatment on an outpatient basis

The types of admissions were similar between the two groups (although no children in the control had been readmitted to treatment). Seven percent of children within the intervention arm were referred from Stabilisation Centres, compared to 6% in the control group. More caregivers referred children to CHWs compared to OTP centres (36.9% vs. 24.7%). As CHWs did not treat SAM in the control group, all children in this group were admitted at health facility level. Meanwhile, 21% of admissions in the intervention group still took place at the health facility level were SAM treatment continued to be available.

**Fig. 2:**
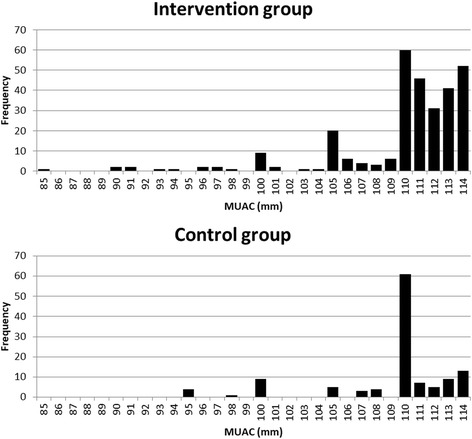
MUAC distribution of children admitted by MUAC in the control area (*n* = 121, Median MUAC = 111 mm) and in the intervention area (*n* = 294, Median MUAC = 111 mm)

Both groups were similar in anthropometric measurements on admission, as can be seen in Table 4, with those in the intervention group had a higher average MUAC than the control group (115.3 mm compared to 113.7 mm in the control group). Further analysis of admission data (see Table 5), showed that 57.8% of children in the intervention group had a MUAC< 110 mm compared to 28.1% in the control group. This was statistically significant (*p* < 0.001, risk ratio = 2.06 [95% CI 1.52; 2.78]; risk difference = 0.30 [95% CI 0.19; 0.39]). There were more cases of oedema at admission in the control group compared to the intervention group (4.5% vs 0.8%), a difference that was statistically significant (*p* = 0.001; risk ratio = 0.18 [95% CI 0.06; 0.51]; risk difference = − 0.04 [95% CI -0.07; − 0.01]). More children were referred to Stabilisation Centres in the control group than the intervention group (5.4% vs 2.6%). Figure 2 shows how children admitted in the intervention group had a MUAC closer to the admission criteria and there is less rounding up in the measurements.

**Table 4:** Anthropometric characteristics of intervention and controls groups at admission

	Intervention (*n* = 699)			Control (*n* = 235)		
	Median	Min	Max	Median	Min	Max
Age (months)	12.0	6.0	59.0	12.0	6.0	48.0
Weight (kg)	6.4	3.2	13.1	6.4	3.1	11.1
Height (cm)	70.1	55.0	103.5	71.0	55.0	86.5
MUAC (mm)	115.0	85.0	150.0	114.0	95.0	150.0

**Table 5:** Analysis of admissions

	Intervention		Control		RR	RD	*p*
	*N*	%	*N*	%	[95% CI]	[95% CI]	
Early Admission							
MUAC at admission< 115 mm	294		121				
(MUAC<=110)	230	78.2	95	78.5	0.99	−0.002	0.999
					(0.89–1.11)	(−0.084–0.089)	
(MUAC> 110)	124	57.8	87	28.1	2.06	0.30	< 0.0001
					(1.52–2.78)	(0.19–0.39)	
New Admissions	621		221				
Oedema	5	0.8	10	4.5	0.18	−0.04	0.001
					(0.06–0.51)	(− 0.07–0.01)	
new cases with complications referred to Stabilization Center on the first day of treatment	16	2.6	12	5.4	0.47	−0.028	0.05
					(0.23–0.99)	(−0.068–0.001)

In relation to the cure ratio, after adjusting for sex, oedema and MUAC at admission the intervention group reported ratios of 94.2% compared to 88.6% in the control group. The risk ratio of 1.89 (95% CI 1.09–3.27) highlights that the probability of being cured was slightly higher in the intervention group (*p* = 0.022). Defaulter ratios were twice as high in the control group compared to the intervention group (10.8% vs 4.5%). The risk ratio of 0.43 (95% CI 0.24–0.77) highlights that the probability of defaulting significantly lower in the intervention group (*p* = 0.005). Within the intervention group, 5 children (0.8%) died compared to 2 children (0.9%) in the intervention group. Given the low ratios in both groups, the difference is not statistically significant (see Table 6).

**Table 6:** Treatment outcomes in intervention group compared to control group, adjusted for sex, oedema and MUAC at admission

	Intervention		Control		RR	*p*
	*N*	%	*N*	%	[95% CI]	
Discharged from OTP	617		212			
Cured	581	94.2	187	88.2	1.89(1.09–3.27)	0.022
Defaulted	28	4.5	23	10.8	0.43(0.24–0.77)	0.005
Died	5	0.8	2	0.9	1.15(0.21–6.05)	0.868
Non respondent	3	0.5	0	0%	NA	NA
Transfers	81		22			
Transferred to another OTP	18	22.2	5	22.7	0.56(0.16–1.96)	0.371
Referred to Stabilisation Centre (SC)	63	77.8	17	77.3	2.25(0.66–7.61)	0.192
*Readmission into OTP when discharged from SC*^a^	51	80.9	11	64.7	NA	NA
*Lost to Follow-Up*	12	19.1	6	35.3	NA	NA

^a^ Outcomes included in “Discharged from OTP”

In December 2014, the treatment coverage in the two groups was estimated to be 43.9% in the intervention group vs 43.8% in the control (*p* value = 0.127). In December 2015, coverage was estimated to be 86.7% in the intervention and 41.6% in the control (*P* value < 0.0001). This difference between the two groups in December 2015 was statistically significant.

## Discussion

This study shows that using CHWs to treat SAM in children without complications is effective and treatment by CHWs has non-inferior outcomes than traditional OTP treatment models. The results highlight that the differences in cure ratios and defaulter ratios were statistically significant. In fact, the relative risk between defaulter ratios in the two groups shows that the likelihood of defaulting was 50% lower in the intervention area than the control.

The sociodemographic baseline survey shows that the two groups are comparable in key health and social indicators. Only roofing type and access to clean water indicators were shown to be different between the control and intervention groups. The choice of construction material for roofing is often a sign of well-being but can also be linked to the customs of each population and physical access to the type of material. Hence, its recognition as a single variable explaining wellness is not recommended. Access to clean water can be linked with the level of coverage of the drinking water distribution network. Theoretically, access to drinking water reduces the prevalence of waterborne diseases, including diarrhea, which has been related to malnutrition. However, evidence for this is minimal as in both areas the prevalence of GAM was very similar in both the intervention and control areas. It is also worth noting that distribution of hygiene kit containing capsules for the treatment of water to each home, if admitted to the program in both areas, minimised the impact of access to clean water. Both areas can therefore be considered comparable for the purposes of this analysis.

Findings from this study are consistent with other research that aimed to evaluate the performance of CHWs treating SAM. In Bangladesh, a 2012 study found the integration of the treatment of SAM into community-based health and nutrition programs to be feasible and effective [17]. However, our study goes one step further by measuring these differences in a routine program setting.

Treatment in both groups met the minimum standards for cure ratio (75%), as outlined in the Mali CMAM Guidelines as well as the SPHERE standards. More children in the intervention were admitted based on Weight for Height criteria: the intervention group shows 53% of children with MUAC between 150 and 115 mm at admission compared to 47% in control. Although this difference wasn’t statistically significant it needs to be further examined, especially given the new national protocols will use MUAC only admission criteria at the community level [21]. This shows that CHWs also use Weight for Height criteria often to admit children into treatment and respect the protocol, despite the fact that this criteria have been considered burdensome and impractical at community level [22]. Differences in oedema at admission were statistically significant (0.7% in the intervention group compared to 4.7% in the control group, *p* = 0.001), this was further observed in coverage and other assessments such as prevalence and surveillance reports. The quality of care for identifying oedema may offer a partial explanation, but the main reason is more likely to be that children identified and treated by CHWs were less likely to have symptoms of oedema at admission due to detection and admission “early” in the SAM episode (i.e. MUAC 114-110 mm) before complications could develop.

Children in the intervention group were also twice less likely to need to be referred to Stabilisation Centres at admission (within 24 h of admission). This, together with oedema at admission and the lower number of referrals on admission, requires further investigation. However, it suggests that SAM treatment by CHWs can lead to children being identified with SAM and reach treatment in a more timely manner than traditional facility-based approaches. However, no difference in the MUAC at admission could be seen between the two groups. This could be the result of “digit preference”, the rounding MUAC measures to the closest number ending in 0 and 5.

The difference in treatment coverage achieved in December 2015 (86.7% in the intervention group compared to 41.6% in the control group) shows that when CHWs offer treatment to children with SAM, more children are able to access treatment services. The differences in self-referrals between the two groups (36.9% vs. 24.7%) highlights that beneficiaries felt confident in allowing CHWs to treat SAM cases. A significant proportion (21%) of all admissions in the intervention area were admitted directly at health facilities. This was reportedly connected to physical proximity between beneficiary communities and the three health facilities, which continued to provide outpatient services in the intervention area. A number of these cases were also detected and enrolled during routine visits to these facilities for other conditions or illnesses. This suggests that facility-based services continue to play an important role in the detection and admission of cases even when CHWs are able to provide treatment in the communities.

Similar progress has been noted with other disease models when treatment has been moved to the community level. In Nepal, a country that has over 20 years of experience in community management of sick children, access to treatment rates reached 69% in children under 5 years old, resulting in a considerable reduction in mortality, due to acute diarrhea and severe pneumonia [11]. In Ghana, 92% of caregivers of sick children sought care from community workers trained in the management of pneumonia and malaria, with most of these children being treated within 24 h from the onset of fever [23]. In Zambia, a study on the integrated management of pneumonia and malaria found that 68% of children with pneumonia received early and appropriate treatment by CHWs [24]. In Ethiopia, the agents deployed in remote communities distributed two and a half times more treatment for the three diseases than all medical units of the same district [23, 25, 26].

The increase in cure ratios within the intervention group reflects the similar reduction in defaulters within this group. Improved access to treatment, reduced travel requirements to health centres and close proximity of community health workers were noted as reasons for the decline in defaulter ratios. No differences among mortality ratios between the two groups were found and both met international SPHERE standards (< 10%) [22].

More children in the intervention group tested positive for malaria (35.9% vs. 19.7%) which may be a reflection of more active care seeking behaviour among caregiver of sick children in the community versus routine check-ups at health facilities. Anthropometric characteristics of intervention and control groups at admission showed no differences, suggesting again that children seeking treatment in both groups were comparable. Similar results were obtained in a study in Bangladesh [27].

This study showed that CHWs are able to treat SAM successfully in field conditions and that such an intervention can lead to improved treatment coverage figures and reduced defaulter ratios. However, this study had a number of limitations that need to be further explored. The study design may have led to some selection bias and caution needs to be exercised when results are extrapolated to broader contexts. The study design used a target population already seeking care in each group, it is therefore possible that the design was less susceptible to recruit difficult to reach cases, though coverage assessments implemented showed that this was limited. While the smaller sample size in the control group was factored into the analysis through ‘matched paired’ analysis, the difference could have still impacted on results. The coverage rates could also be influenced by an intervention bias- the multiple research and surveys implemented may raise awareness of the program, potentially leading to increased coverage rates. The study area was supported by an international NGO and hence, may reflect performance levels associated with well-supported interventions.

## Conclusion

This study suggests that with minimal training, CHWs are able to integrate SAM treatment into the iCCM package of care. Allowing CHWs to treat SAM reduces defaulter ratios without compromising outcomes of treatment (cure ratios and mortality ratios remain similar to that of a traditional OTP approach). The model was positively received by the community, with an increased number of self-referrals to treatment being seen in the intervention group. Furthermore, coverage rates highlight that using CHWs to treat SAM can lead to improved access to treatment. Hence, the model has been shown to be effective and should be explored in other contexts. Future research should explore; a) the implications of adding SAM treatment on the quality and coverage of other services provided by CHWs (malaria treatment, pneumonia etc.); b) the viability of achieving similar results at scale; c) the impact of different types of CHW supervision, and; d) the impact of CHW-delivered treatment on non-response, relapse and the mortality and morbidity outcomes amongst defaulters.

## Abbreviations

CHW: Community health worker
CMAM: Community-based management of acute malnutrition
CTC: Community-based therapeutic care
iCCM: Integrated community case management [of childhood illnesses]
MAM: Moderate acute malnutrition
NGO: Non-Governmental Organisation
SAM: Severe acute malnutrition
SQUEAC: Semi-Quantitative evaluation of access and coverage
